# Pigment Production on L-Tryptophan Medium by *Cryptococcus gattii* and *Cryptococcus neoformans*

**DOI:** 10.1371/journal.pone.0091901

**Published:** 2014-04-15

**Authors:** Stuart Chaskes, Michael Cammer, Edward Nieves, Arturo Casadevall

**Affiliations:** 1 Department of Biology, Farmingdale State College, State University of New York, Farmingdale, New York, United States of America; 2 Microscopy Core, New York University Langone Medical Center, New York, New York, United States of America; 3 Laboratory for Macromolecular Analysis & Proteomics, Albert Einstein College of Medicine, Bronx, New York, United States of America; 4 Department of Microbiology and Immunology, Albert Einstein College of Medicine, Bronx, New York, United States of America; 5 Department of Medicine, Albert Einstein College of Medicine, Bronx, New York, United States of America; Massachusetts General Hospital, Harvard Medical School, United States of America

## Abstract

In recent years strains previously grouped within *Cryptococcus neoformans* have been divided into two species *C. neoformans* and *C. gattii*, with *Cryptococcus neoformans* comprising serotypes A, D, and AD and *C. gattii* comprising serotypes B and C. *Cryptococcus neoformans* have also been subdivided into two varieties *C. neoformans* var. *grubii*, serotype A, and *C. neoformans* var. *neoformans,* serotype D. We analyzed the growth and pigment production characteristics of 139 strains of *Cryptococcus* spp. in L-tryptophan containing media. Nearly all strains of *Cryptococcus,* including each variety and serotype tested produced a pink water-soluble pigment (molecular weight of 535.2 Da) from L-tryptophan. Consequently, the partial separation of the species was based on whether the pink pigment was secreted into the medium (extracellular) or retained as an intracellular pigment. On L-tryptophan medium *C. neoformans* var. *grubii* and serotype AD produced a pink extracellular pigment. In contrast, for *C. gattii,* the pink pigment was localized intracellularly and masked by heavy production of brown pigments. Pigment production by *C. neoformans* var. *neoformans* was variable with some strains producing the pink extracellular pigment and others retained the pink pigment intracellularly. The pink intracellular pigment produced by strains of *C. neoformans* var. *neoformans* was masked by production of brown pigments. *Cryptococcus* laccase mutants failed to produce pigments from L-tryptophan. This is the first report that the enzyme laccase is involved in tryptophan metabolism. Prior to this report *Cryptococcus* laccase produced melanin or melanin like-pigments from heterocyclic compounds that contained ortho or para diphenols, diaminobenzenes and aminophenol compounds. The pigments produced from L-tryptophan were not melanin.

## Introduction

In the late 1990s *Cryptococcus neoformans* was subdivided into the three varieties: *C. neoformans* var. *gattii* (serotypes B and C), *C. neoformans* var. *neoformans* (serotype D), *C. neoformans* var. *grubii* (serotype A) and *C. neoformans* (serotype AD) [1]. Since then, *Cryptococcus* was further divided into two species; *C. gattii* and *C. neoformans* with the latter including two varieties (C. *neoformans* var. *neoformans* and *C. neoformans* var. *grubii)* [2]. *C. neoformans* var. *grubii, C. neoformans* var. *neoformans* and *C. gattii* cause disease in immunocompromised patients [3]–[8], whereas *C. gattii* remains a pathogen primarily for individuals with no known immunological deficit [4], [8]. Recent outbreaks and surveillance of the emerging pathogen *C. gattii* have occurred on Vancouver Island in Canada [9]–[11], the United States [12] and Africa [3], [5], [7]. Consequently, it is important to be able to distinguish *C. gattii* from *C. neoformans* since there are clinical differences in cryptococcosis caused by these two pathogens [11]–[16].

Pigment production has historically been an important aid in the isolation, classification, and identification of the clinically important yeast [17]–[19]. *C. neoformans* produced melanin from 3, 4-dihydroxyphenylanine (DOPA), and other o- and p-diphenols [17]–[30]. Melanin like-pigments were also synthesized by this yeast from aminophenols [25], [31], diaminobenzenes [25], [31], and indole derivatives with a hydroxyl or an amino group on the phenyl ring [32]. The enzyme laccase metabolized these substrates to melanin which was deposited in the cell wall of *Cryptococcus* spp. [33], [34].

*Cryptococcus neoformans* and *Candida albicans* both produced a pink extracellular pigment when cultured on L- or DL- tryptophan medium [35], [36]. However, the cryptococcal serotypes used in that study were not known. In subsequent studies we found that the D-enantiomer form of tryptophan was also converted to the pink pigment by *C. albicans* [37]. In contrast, *C. gattii* and *C. neoformans* failed to produce the pink pigment from D-tryptophan [37]. Ability to grow on minimal D-tryptophan D- proline agar (m-DTDP) differentiates *C*. *gattii* from *C. neoformans* var. *neoformans* and *C. neoformans* var. *grubii* [37]. *C. gattii* isolates grew on m-DTDP and produced brown pigments whereas *C. neoformans* var. *grubii* and *C. neoformans* var. *neoformans* failed to grow since they do not utilize the D- amino acids [37]. The melanin and melanin-like pigments are distinct and not directly related to the tryptophan derived pigments [37].

A major goal of this study was to determine which *Cryptococcus* species and serotypes produced the pink water soluble pigment from L-tryptophan and which produced brown pigments from L-tryptophan. Another major goal was to determine whether the pink water soluble pigment produced by *Candida albicans* and *Cryptococcus neoformans* were identical and to determine the role of laccase in pigment production from L-tryptophan. Secondary goals were to partially characterize the pigments that were produced from L-tryptophan. We report here that *Cryptococcus* spp. metabolized tryptophan by a synthetase pathway to produce pigments and fluorescent metabolites with a molecular mass larger than L-tryptophan. Our results indicate that significant metabolic differences exist in the metabolism of L-tryptophan by *Cryptococcus* species.

## Material and Methods

### Cultures

This study utilized yeasts that were previously described; *C. gattii* (67 strains), *C. neoformans* var. *grubii* (33 strains), *C. neoformans* var. *neoformans* (25 strains), and serotype AD (14 strains) [37].The cryptococcal laccase mutants [38], [39] used in this study included 2ETU (laccase partial deletion mutant) and 2ETU-C (complemented strain), obtained from June Kwon-Chung, (National Institutes of Health, Bethesda, MD), and MDJ12 (*lac1* mutant), QGC8 (*lac1* and *lac2* double mutant), and RPC26 (*lac2* mutant) obtained from Joseph Heitman and J.A. Alspaugh, (Duke University, Durham NC). *C. albicans* (BSMY 212) was provided by David Goldman, (Albert Einstein College of Medicine, New York, NY).

### Inoculums

Two- to 5 day old yeast cells from Sabouraud dextrose agar plates were transferred to quad Petri plates using a 10-ul inoculating loop. The plates were inoculated so that confluent growth developed. Broth cultures were inoculated with one 10-ul loopful of *Cryptococcus* sp. per 250 ml L-tryptophan medium.

### Conditions for Pigment Production of *Cryptococcus* spp. on L-tryptophan Agars

The pH was adjusted to 5.35 in all tryptophan media since previous studies of *C. neoformans* and *C. albicans* had shown that pink pigment production occurred only below pH 5.5. D and L-tryptophan alone does not support the growth of *Cryptococcus* and *Candida* spp. in a chemically defined medium [31], [35], [37]. Therefore, the following auxiliary nitrogen sources were tested at 0.5 and 1 g/liter: glycine, L glutamine, L-serine, L-proline, L-asparagine and ammonium sulfate to determine if they supported pink pigment production from tryptophan. L-Tryptophan concentrations tested were: 1, 2, and 5 g/liter. The following additional variations were included to determine the optimal conditions for pink pigment production: incubation temperatures of: 22°, 25°, 28°, 30°, 33°, and 37°C: glucose or fructose concentrations of 5, 20, 40 and 80 g/liter.

The influence of laboratory lighting on pigment production was also determined. Previous data with both *Candida* and *Cryptococcus* indicated that light influences pink pigment formation [31], [35], [37]. The L-tryptophan agar plates were incubated without light, with fluorescent ceiling light, and with a fluorescent lamp (28 watt) that was placed 6 to 18 inches above the cultures. The distance of the light source was adjusted so that the temperature of the L-tryptophan plates remained at the desired temperature. The standard fluorescent light includes the 400 to 490 nm range.

### Minimal L-tryptophan Glycine (m-LTG) Medium

Minimal L-tryptophan glycine agar (m-LTG) contained the following: 2X chemicals were prepared in 500 ml dH_2_O: 5.0 g glucose or fructose, 5 g L-tryptophan, 0.5 g glycine, 4 g KH_2_PO_4_, 2.5 g MgSO_4_.7H_2_O, and 10 mg thiamine hydrochloride. The 2X chemical solution was adjusted to a pH 5.35 and filter sterilized. Five hundred ml of 3% agar solution was autoclaved and 500 ml of the 2X chemical solution was added to the sterile agar solution. The poured m-LTG agar Petri quad plates were colorless. Broth shake cultures were grown at 30°C while shaking at 150 rpm.

### Minimal Fructose L-tryptophan Glycine (m-FLTG) Medium

Minimal fructose L-tryptophan glycine agar (m-FLTG) contained the following: 2X chemicals were prepared in 500 ml dH_2_O: 40 g fructose or glucose, 2 g L-tryptophan, 0.5 g glycine, 4 g KH_2_PO_4_, 2.5 g MgSO_4_.7H_2_O and 10 mg thiamine. The 2X chemical was adjusted to pH 5.35 and filter sterilized. Five hundred ml of 3% agar solution was autoclaved and the 2X chemical solution was added to the sterile agar solution. The poured m-FLTG agar Petri quad plates were colorless. Broth shake cultures were grown at 30°C while shaking at 150 rpm.

### Production of the Pink Pigment by *C. Albicans*

The L- or DL- tryptophan media of Chaskes and Phillips [35] and the D- tryptophan medium of Chaskes et al [37]; minimal D-tryptophan D-proline agar (m-DTDP) or minimal Fructose D-tryptophan glycine agar (m-FDTG) were used to produce the pink pigment after exposure to light.

### Partial Purification of the Extracellular Pink Pigment

Two week old cultures of *C. neoformans*, (H99, serotype A), grown in m-LTG or m-FLTG broth, and two week old cultures of *C. albicans* (BMSY 212) grown on L- and D-tryptophan media, were centrifuged at 3000 g for 15 minutes and the supernatant was sterilized by filtration. A 25 to 50 ml of culture supernatants was lyophilized and the resulting residue was dissolved in 5 ml of methanol. Alternately, 5 ml of n-butanol was added to 100 ml of sterile supernatant and vortexed vigorously. The pink pigment was concentrated in the butanol layer (top) which was removed from the water layer. The alcohol extracted pink pigment was subjected to thin layer chromatography (TLC) on silica gel 60 plates (Selecto Scientific, Atlanta, GA), using the solvent system consisting of butanol, ethanol, and water at a 4∶1∶1; v/v/v. After air drying, the pink pigment spots were scraped off and dissolved in 50 percent methanol. The suspension was then centrifuged at 12,000 g to separate the solution from the particulate material from the silica gel matrix. The pink pigment was then acidified with one drop of 1 N HCL and the sample was analyzed by mass spectrometry. A duplicate TLC plate was sprayed with 1% ninhydrin in 100 ml ethanol.

The pink extracellular pigment was also partially purified using a C18 column (Sep-Pak C18, cartridge, Waters, Milford, MA). The column was first washed with 8 ml of 100% methanol (pH 2.5). Twenty- five to 50 ml of lyophilized supernatant was then dissolved in 5 ml methanol. One-half ml of the methanol solution of pink pigment was applied to a C18 column. The column was washed repeatedly with 20% methanol (pH 2.5) to remove L-tryptophan and other non-pigmented compounds. The washing continued as long as the fractions fluoresced under a UV lamp at either 254 or 365 nm. The pink pigment was then eluted off of the column with two 5 ml volumes of 40% methanol (pH 2.5), followed by two volumes of 45% methanol, and two volumes of 50% methanol.

### Fluorescence of *Cryptococcus* spp. Grown on L-tryptophan Media under a UV Lamp

The natural fluorescence of *C. neoformans* and *C. gattii* cells growing in L- tryptophan (m-LTG, and m-FLTG, was studied under a UV lamp at 365 and 254 nm.

### Visible Spectra of the TLC Eluted Extracellular Pink Pigment

The absorbance spectra of the pink pigment were scanned at pH 4 to 5 from 400–750 nm using a spectrophotometer (Bio-Rad Laboratories Smartspec 3000, Hercules, CA). The scan was repeated after adjusting the pH to 1 with 2N HCL. The pink pigment was then scanned a third time after the pH was adjusted to 12 with 2N NaOH. The control consisted of extracts of TLC silica scrapings taken from an area where the compounds were not detected. The control absorbance readings were also pH adjusted and automatically subtracted from the various scans.

### Light and Fluorescent Microscopy

Light and fluorescent microscopy of cryptococcal cells after growth in tryptophan media (m-LTG and m-FLTG) were performed as previously described [37]. Briefly, 2 day to 2 week old cells were centrifuged at 3000 g for 60 minutes, and the pellets were washed in dH_2_O or phosphate buffered saline (PBS) one or two times. Yeast cells were suspended in PBS and mounting fluid and observed with an Olympus AX-10 fluorescent microscope using fluorescein isothiocyanate (FITC) (excitation 480 nm, emission 535 nm), and or Rhodamine filter (excitation 535 nm, emission 610 nm) mode and filters. The yeast cells were also observed under visible light.

### Mass Spectrometry of Pigments

The pink extracellular pigment produced by *C. albicans* from both L-and D-tryptophan and the pink extracellular pigment produced by *C. neoformans* var. *grubii, C. neoformans* var. *neoformans,* and *C. gattii* were eluted from a TLC plate and dissolved in 50% methanol. Mass spectrometric measurements of the pink pigment were performed on a LTQ linear ion trap mass spectrometer (LTQ, Thermo, San Jose, CA) using electrospray positive ionization. The samples were diluted into 50% methanol: water (1∶10 v/v) and infused into the mass spectrometer at a flow rate of 3 µL/min. Tandem mass spectrometry (MS/MS) was performed using an isolation width of 1.5 *m/z* and a normalized collision energy of 25–35%. Several additional pigments and fluorescent compounds were eluted from a TLC plate, dissolved in 80% methanol and the molecular mass of each compound was determined.

### Detection of Indole and Aromatic Compounds

Various Salkowski and Kovacs reagents were made as previously described [37]. One ml of supernatant containing m-LTG, or m-FLTG, was tested with 9 ml of various Salkowski reagents. One-half milliliter of Kovacs reagent was added to 3 ml of the various supernatants.

### Extraction of Intracellular Brown and Pink Pigments

Two week old cultures containing m-FLTG broth of *C. gattii* (serotypes B and C) and *C. neoformans* var. *neoformans* were centrifuged at 3000 g for 60 minutes. The supernatant was sterilized by filtration and the pellet (5 ml of cells) was extracted with 100% methanol (10 ml). The extraction was repeated 3 times and the pooled samples were centrifuged at 10,000 g for 10 min. The soluble extract was concentrated by evaporation in a Petri plate. The sample was resuspended in 2 ml methanol. A second procedure using 2 week old yeast cells extracted the 5 ml cell pellet once with 5 ml of 100% methanol. A third procedure extracted 2 to 3 day old yeast cells. The alcohol soluble pigments (extracted from m-FLTG or m-LTG grown cells) were spotted on TLC Silica gel 60 plates and then examined under a UV lamp at 254 and 365 nm. The solvent system contained either 75% methanol or butanol, ethanol, and water (4∶1∶1; v/v/v). Several different fluorescent compounds and a pink water insoluble pigment were scraped from the TLC plate and dissolved in 100% methanol. The sample was centrifuged at 12,000 g for 5 minutes to remove the silica gel. The compounds were then analyzed in the mass spectrophotometer.

### Thin-Layer Chromatography (TLC)

The 100% methanol extracts containing brown and pink pigments were spotted on TLC silica gel plates as previously described [37]. The solvent system was 75% methanol at either pH 7 or pH 4 to 5. The pink extracellular concentrated supernatant was separated using a solvent system of butanol: ethanol: water (4∶1∶1; v/v/v). The pink extracellular pigment eluted from a C18 Sep-Pak column was separated via TLC with a solvent consisting of 100% methanol at pH 5.

### Transmission Electron Microscopy

*C. gattii* (NIH 112) cells were cultured for one to two weeks on m-FLTG medium. Control cells were cultured on a medium in which glutamine was substituted for tryptophan. The control cells were devoid of pigment. Yeast cells were fixed with 2.5% glutaraldhyde in a 0.1 M sodium cacodylate buffer, post-fixed with 1% osmium tetroxide followed by 1% uranyl acetate, dehydrated through a graded series of acetone and embedded in Spurr’s resin (Electron Microscopy Sciences, Fort Washington, PA). Ultra-thin sections were cut on a Reichert Ultracut UCT, stained with uranyl acetate followed by lead citrate, and viewed on a JEOL 1200EX transmission electron microscope at 80 kv. The normal dehydration reagent (alcohol) was not employed since it extracted the brown pigments from *C. gattii*.

### Isolation of L-tryptophan Particles

*C. gattii* cells were grown in m-FLTG broth for 3 weeks. Yeast cells were centrifuged for 60 minutes at 3,000×g. The cells were washed in 0.85% saline (3 to 5x) until the supernatants were clear. The procedure of Wang et al. [40] that was used to make melanin particles (“ghosts”) was adapted to *C. gattii* cells which formed brown pigment in L-tryptophan broth (m-FLTG).

## Results

### Conditions for Pigment Production of *Cryptococcus* spp. on L-tryptophan Agars

Our first goal was to optimize pigment production by *Cryptococcus* spp. on tryptophan-containing agar by evaluating the effect of other amino acids as a supplemental nitrogen source. L-proline, L-serine, and ammonium sulfate were eliminated as possible sources of auxiliary nitrogen since they failed to support pink pigment production from L-tryptophan by *C. neoformans*. In contrast, glycine, L-asparagine, and/or L- glutamine did support pink pigment production from L-tryptophan. Glycine was selected for further studies because under ordinary laboratory lighting it induced the formation of the pink pigment. Additionally, more strains produced the pink pigment with glycine as the auxiliary nitrogen source than with any other amino acid (data not shown). With glycine all strains of *C. neoformans* var. *grubii* produced the pink pigment under normal laboratory lighting, and most strains produced some pigment in the dark. L-asparagine or L- glutamine was not used because they required the positioning of a fluorescent light 6 to 18 inches above the plates for induction of pink pigment production.

Next we optimized the carbohydrate concentration. The glucose or fructose concentration was set at 5 g/liter (m-LTG) to maximize the number of strains that produced the pink pigment. At higher sugar concentrations some strains produced either smaller amounts or failed to produce the pink pigment at all. However, the strains that produced large amounts of the pink pigment at a 5 g/liter sugar concentration produced copious amount of the pink pigment at 40 g/liter (m-FLTG). Production of the extracellular pink pigment required placing a fluorescent lamp 6 to 18 inches above the m-FLTG plates after 3 days growth (Table 1). Pink pigment production was consistent using either glucose or fructose as the carbohydrate source.

**Table 1:** Optimum conditions for the production of the extracellular pink and intracellular brown pigments formed by *Cryptococcus* spp. from L-tryptophan.

Condition Tested	Extracellular Pink Pigment^a^	Intracellular Brown Pigment^a^
I. Carbohydrate Concentration		
A.5 g/l glucose or fructose	++++	++
B. 40 g/l glucose or fructose	++	++++
II. Light (400–490 nm)		
5 g/l glucose or fructose	Minimum Light required	Light not required
40 g/l glucose or fructose	Maximum Light required	Light not required
III.L-tryptophan Concentration		
5 g/l	++++	++
2 g/l	++	++++
IV. Supplemental Nitrogen Source		
Ammonia or L-serine or L-proline	0	0
Glutamine or asparagine	+++	++
Glycine	++++	++++
V. pH		
5.35	++++	++++
7.00	0	++++
VI. Temperature		
20°C	++	++
28–30°C	++++	++++
33–37°C	0	++++

a A scale of 0 to ++++ was used. 0 no pigment formation ++ adequate pigment formation, ++++ excellent pigment formation.

The temperature range for pink pigment production was 22° to 30°C with the optimum at 28° to 30°C. In the temperature range of 33° to 37°C there was either a dramatic decrease or a complete failure to produce the pink pigment. The extracellular pink pigment was produced by *C. neoformans* when the pH was set below 5.5.

*C. gattii* produced ample amounts of brown pigments from m-LTG medium. The amount of brown pigment produced was significantly increased at higher glucose or fructose (m-FLTG) concentrations (40 to g/liter). *C. gattii* produced a greater quantity of brown pigments, as determined visually, from fructose as compared to glucose. The production of the brown pigments by *C. gattii* was neither pH nor temperature sensitive. The brown pigments were produced at pH and temperature ranges from 5 to 7 and at 20° to 37°C, respectfully. Table 1 summarizes the optimum conditions for production of the pink extracellular and brown intracellular by *Cryptococcus* spp. These results led to the formulation of m-LTG medium for maximum production of the extracellular pink pigment and m-FLTG medium to maximize intracellular brown pigment formation. The conditions for pink pigment production were more stringent than for brown pigment formation (Table 1). The m-LTG medium gave the best results for both pink and brown pigment production.

### Growth and Pigmentation of *C. Neoformans* Var. *Grubii, C. Neoformans* Var. *Neoformans,* and *C. Gattii* on m-LTG Agar

**Figure 1:**
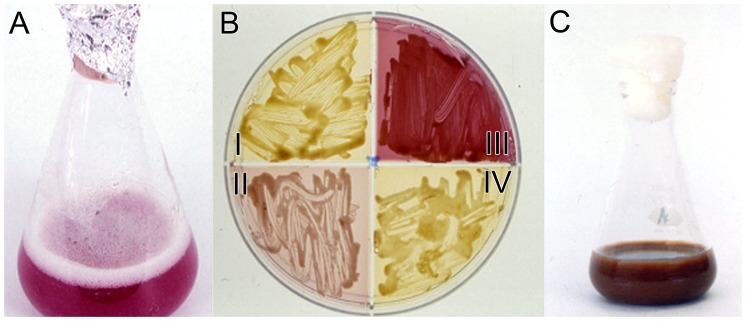
Pink and brown pigment production by *Cryptococcus*. The intensity of the pink and brown pigments were estimated visually. Strong pink pigment was produced in m-LTG media by *C.neoformans* var. *grubii* (A) *C. gattii* (B) formed light brown pigments on quadrants I and IV and *C. neoformans* var. *neoformans* and *C. neoformans* var. *grubii* produced pink pigments (quadrants II and III) respectively on m-LTG agar. The intensity of the pink pigment varied from strain to strain. Some strains of *C. neoformans* var. *neoformans* produced brown pigments on m-LTG agar (not shown). *C. gattii* produced an intense brown pigment after growth in m-FLTG medium (C).

At 30°C and pH 5.35 m-LTG agar (Table 2) optimizes pink extracellular pigment production by *C. neoformans* var. *grubii, C. neoformans* var. *neoformans* and serotype AD. One hundred percent (33/33) of *C. neoformans* var. *grubii* strains grew at 22° to 30°C, and produced the pink extracellular pigment on m-LTG agar (Fig. 1A and 1B). Approximately 85% of the *C. neoformans* var. *grubii* isolates produced the pink pigment after 2 to 5 days growth. The remaining 15% produced smaller amounts that became visible after 4 to 7 days growth. Eighty six percent (12/14) of *C. neoformans* (serotype AD) strains produced the pink extracellular pigment. Brown pigment production was not observed for serotype AD under any condition. The two strains of serotype AD strains that failed to produce the pink pigment were unusual since they were the only strains that formed an orange pigment after four or five days on Sabouraud Dextrose agar. Ninety-nine percent (66/67) of *C. gattii* strains produced brown intracellular pigments on m-LTG agar (Table 2, Fig. 1B). One strain produced only a trace of the brown pigments. Pigment production was evident after 2 to 5 days incubation at 22° to 30°C. Pigmentation continued to increase over the next two or three weeks. The greatest variation of pigment production from L-tryptophan was observed with *C. neoformans* var. *neoformans.* Thirty-six percent (9/25) of *C. neoformans* var. *neoformans* strains produced the pink extracellular pigment, 52 percent (13/25) produced the brown pigments, and 12 percent (3/25) produced small amounts of the brown pigment after 10 days incubation (Table 2). Occasionally, a few strains of *C. neoformans* var. *neoformans* produced both the pink extracellular pigment and brown intracellular pigments simultaneously. Those strains that produced both pigments usually produced small quantities of the extracellular pink pigment.

**Table 2:** Comparison of pigment production from L- and D-Tryptophan by *Cryptococcus* spp.

	L-Tryptophanm-LTG or m-FLTG agar	D-Tryptophanm-DTDP agar	D-Tryptophanm-FDTG agar	D-Tryptophan References
*C. gattii*	Dark brown^a^ (m-FTLG) Light brown (m-LTG) intracellular pigments (99%)	Dark brown^a^ intracellular pigments	Dark brown^a^ intracellular pigments	37,56
*C. neoformans* var. *grubii*	Pink extracellular Pigment (100%)	No growth	Light brown intracellular pigments	37
*C. neoformans* var. *neoformans* b ^,^ c	Brown intracellular pigments (64%)^a^ Pink extracellular pigment (36%)	No growth	Light brown intracellular pigments	37

a Dark Brown is the color of the pigmentation observed. The dark brown pigment masks pink and yellow intracellular pigments that were also produced and detected by TLC.

b A few strains produced both the pink extracellular and brown intracellular pigments.

c One strain failed to grow on m-LTG and m-FLTG agar.

Two of the *C. neoformans* var. *neoformans* isolates exhibited both a smooth and mucoid colony type. The smooth type produced the pink pigment and the mucoid types the brown pigments for the first set whereas the pattern was reversed for the second set.

The results for three strains of *Cryptococcus* in which the genome has been sequenced were as follows: *C. neoformans* var. *grubii* (H99) produced the pink pigment and *C. neoformans* var. *neoformans* (JEC 21 and B3501) produced brown pigments. Table 2 summarizes the results of the *Cryptococcus* spp. on m-LTG agar.

### Growth and Pigmentation of *C. Gattii* and *C. Neoformans* on m-FLTG

m-FLTG differs from m-LTG by having higher sugar and lower L-tryptophan concentrations. m-FLTG maximized production of the brown pigments by *C. gattii* by a factor of 2–3x as assayed visually relative to m-LTG agar. This medium was primarily designed for *C. gattii* to produce copious amounts of the brown pigments (Fig. 1C). The strains of *C. neoformans* var. *grubii* and *C. neoformans* var. *neoformans* that produced large quantities of the extracellular pink pigment on m-LTG agar produced even larger quantities of the pigment on m-FLTG agar. However, the time required for pink pigment production was sometimes 7 days and intense coloration required up to two to three weeks for some strains. Furthermore, some strains required light stimulation for the production of the extracellular pink pigment on m-FTLG agar.

### Fluorescence of *Cryptococcus* spp. Grown on L-tryptophan Media under a UV Lamp

Previous results of *C. gattii* cultured on D-tryptophan agars resulted in the formation of strong fluorescence after exposure to UV light at 365 nm [37]. *C. gattii* strains cultured on m-FLTG agar were highly fluorescent at 365 nm after one to three days growth. The initial fluorescence was quenched as soon as the *C. gattii* strains produced large amounts of the brown intracellular pigments. *C. neoformans* var. *neoformans* strains that produced the brown pigments were also fluorescent after 1 to 3 day’s growth and their fluorescence was also quenched after the production of the brown pigments. Weaker fluorescence was observed when *C. gattii* was cultured on m-LTG agar. These results were not shown since they are identical to the results obtained with D-tryptophan [37]. In contrast, the yeasts which produced the extracellular pink pigment from L-tryptophan medium, *C. neoformans* var. *grubii, C. neoformans* var. *neoformans,* and serotype AD were either not fluorescent or were weakly fluorescent at 365 nm. Fluorescence was absent or minimum for all *Cryptococcus* spp. at 254 nm.

### Visible Spectra of the TLC Purified Pink Pigment

**Figure 2:**
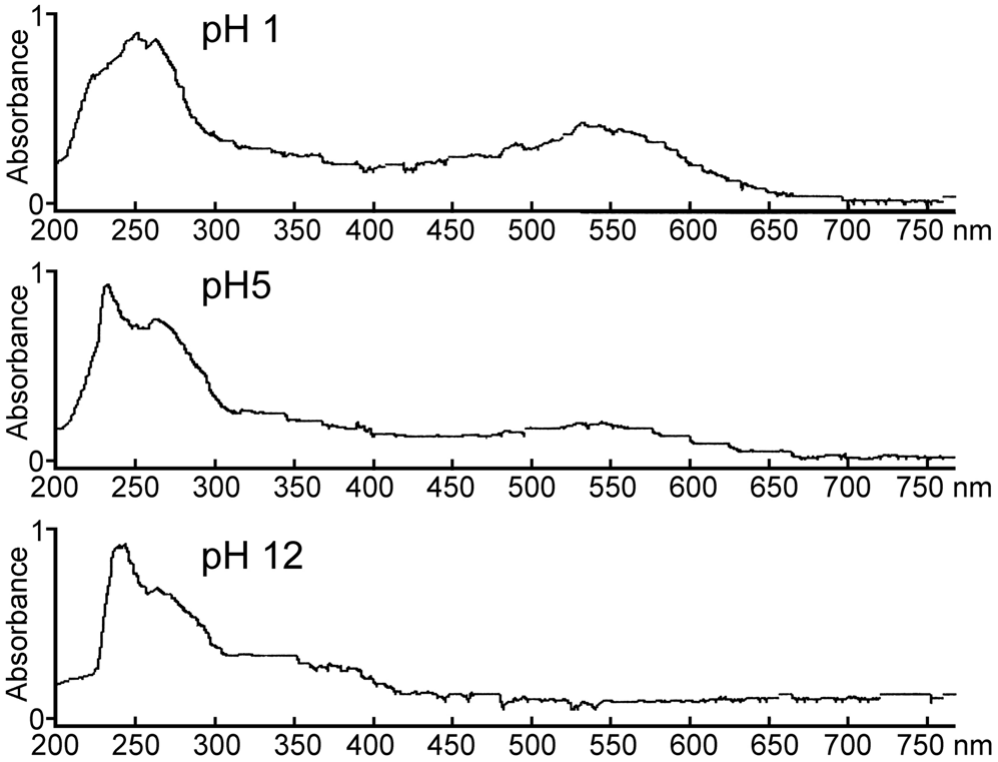
Pink pigment spectra from 200 to 760 nm. The scan at pH 5 of the purified pink pigment revealed two peaks in the UV range (231 and 265 nm). A single peak was seen in the visible range (538 nm). The scan at pH 1 shows a shift in the UV peaks to (250 and possible 260 nm) and a single enhanced peak (at 538 nm). The scan at pH 12 reveals a UV peak at 243 nm which had a large shoulder and the peak at 538 nm has disappeared. The disappearance of the peak at 538 nm corresponded to the pink pigment becoming colorless above the pH of 6.0.

The pink extracellular pigment produced by *C. neoformans* var. *grubii*, *C. neoformans* var. *neoformans* and *C. albicans* (from L- tryptophan) was a pH indicator. The pink color was stable below pH 5.5; faded at pH 5.6 and it disappeared at pH≥6.0 (Fig. 2). The pink coloration reappeared if the pH was again adjusted below 5.5 even after the solution was alkalinized to pH 12. The intensity of the pink color increased approximately two fold if the pH was lowered from 5.5 to 1. The maximum visible absorption wavelength for the pink pigment was between 535 and 540 nm at both pH 1 and 5 (Fig. 2).

### Light and Fluorescent Microscopy (FITC Filter)

**Figure 3:**
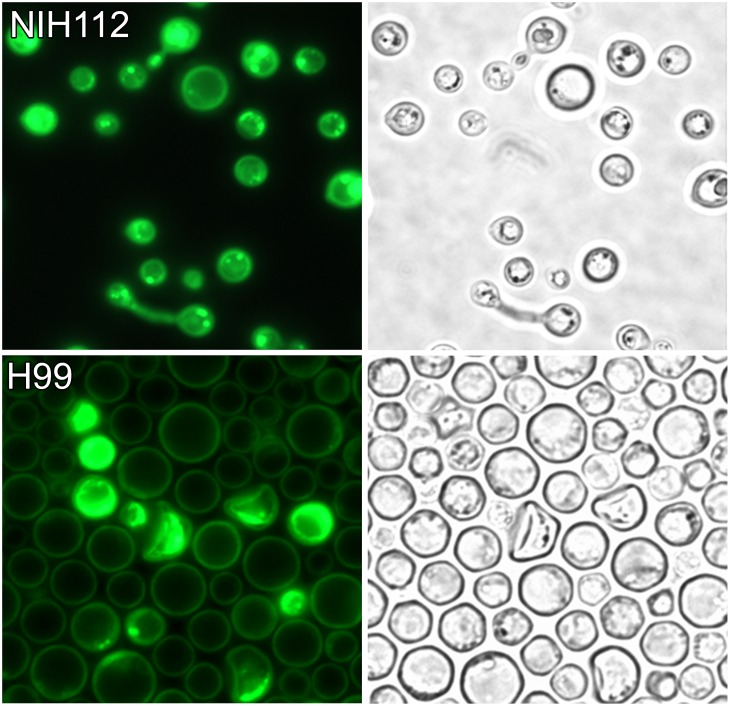
Visible and fluorescent microscopy of *Cryptococcus.* Visible and fluorescent microscopy (FITC filter) of *C. gattii,* NIH 112, (top) and *C. neoformans* var. *grubii,* H99, (bottom). The majority of the yeast cells have a normal appearance but atypical morphology was occasionally observed after growth on tryptophan. *C. gattii* and *C. neoformans* var. *grubii* cells exhibit strong fluorescence with the FITC filter. *C. gattii* cells often contained small concentrated foci that were fluorescent. The cell walls of *C. neoformans* var. *grubii* were usually fluorescent. The strains of *C. neoformans* var. *neoformans* which produced brown pigments exhibited a fluorescent pattern that resembled *C. gattii* whereas the strains that produced the pink pigment had a fluorescent pattern that was similar to *C. neoformans* var. *grubii.*

*C. gattii* (Fig. 3, NIH112 top), and *C. neoformans* var. *grubii* cells (Fig. 3, H99 bottom) fluoresced after growing on L-tryptophan medium. The fluorescence was observed for cells in the stationary phase of growth (4 to 12 days) whereas fluorescence was very weak or negative for cells in the log phase of growth (1 to 2 days). *C. neoformans* var. *neoformans* and serotype AD were fluorescent with the FITC filter (results not shown). The great majority of cryptococcal cells exhibited normal morphology. Occasionally short pseudohyphae and germ tube formation were observed on tryptophan medium (Fig. 3, top).

### Mass Spectrometry Analysis

**Figure 4:**
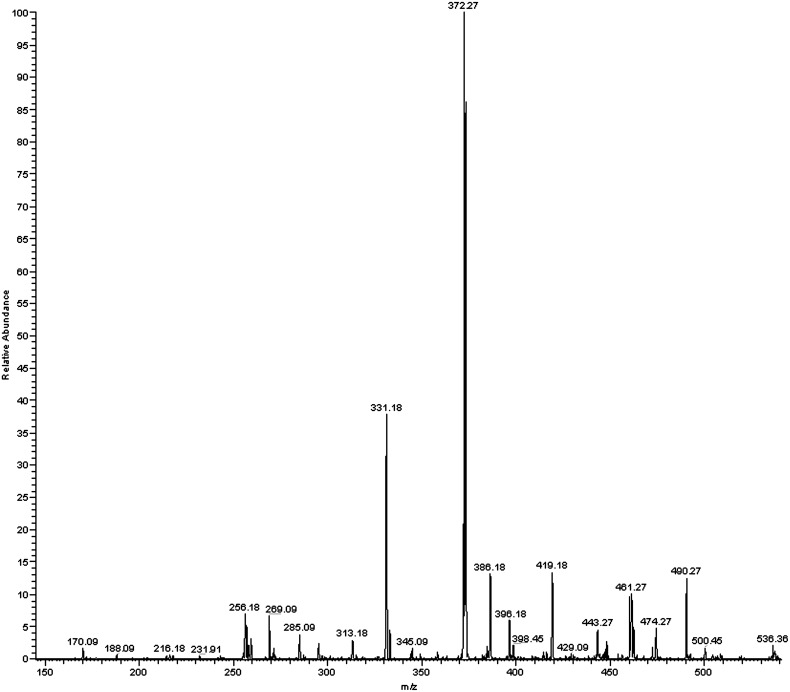
Tandem mass spectrometry. Tandem mass spectrometry (MS/MS) of precursor ion 536.2 *m/z* using an isolation width of 1.5 *m/z* and normalized collision energy of 25–35%.

The extracellular pink pigment produced by *Cryptococcus s*pp. from L-tryptophan had a mass of 535.2 Da. Tandem mass spectrometry (MS/MS results of the precursor ion 536.2 were obtained (Fig. 4) producing a major product ion of 372.3 *m/z.* Identical results were obtained when the pink pigment was produced by *C. albicans* from both L- and D-tryptophan.

### Detection of Indole and Aromatic Compounds

Kovacs reagent did not detect indole or closely related indole derivatives in the supernatants of *C. gattii, C. neoformans* var. *grubii, C. neoformans* var. *neoformans* and serotype AD. Salkowski reagent has a wider specificity for detecting aromatic ring compounds than Kovacs reagent. The supernatants of *C. gattii* (light brown) and the supernatants of *C. neoformans* var. *neoformans* that produced brown pigments reacted with Salkowski reagent to form a pink to fuchsia color. These results were identical to those reported when D-tryptophan medium was used [37]. Salkowski reagent did not react with the pink pigment containing supernatants. The un-inoculated medium did not react with Salkowski reagents.

### Extraction of Intracellular Brown and Pink Pigments

Methanol was the preferred solvent to extract pigments and fluorescent compounds from *C. gattii* and *C. neoformans* var. *neoformans* after growth in L-tryptophan medium. Alternately ethanol or *n*-butanol was able to extract these compounds. Colored and fluorescent photographs of the alcohol extracted pigments were not shown since the results were identical to those obtained with D-tryptophan [37].The brown pigments masked fluorescence at 365 nm in water based medium. However, the fluorescence returned once the yeast cells were extracted with alcohol. The brown pigments and fluorescent compounds could not be extracted from these yeasts with 100% chloroform, acetone, acetonitrile, or xylene.

### Thin-Layer Chromatography (TLC)

**Figure 5:**
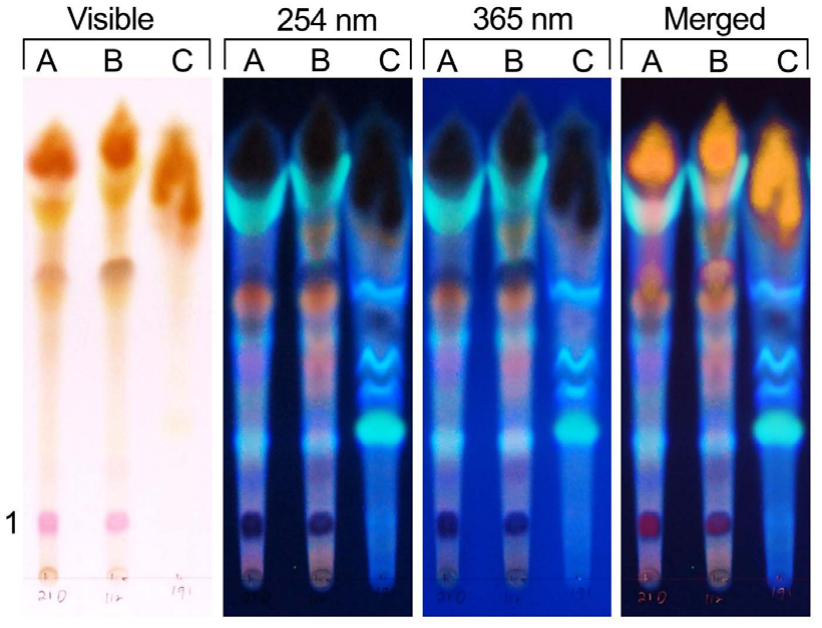
Extracted pigments and fluorescent compounds of *Cryptococcus* using large volumes of methanol. The methanol extracted pigments and fluorescent compounds of *C. neoformans* var. *neoformans* (column A), *C. gattii*, serotype B, (column B) and *C. gattii,* serotype C (NIH 191, column C). The pink water insoluble pigment (1) was produced by nearly all of the strains tested except for NIH 191, serotype C. The repeated methanol extracts removed most of the brown pigments. NIH 191 was selected because it shows considerable variations from many of the other strains. NIH 191 produced multiple fluorescent compounds that were not produced by most strains of *C. gattii.* The solvent system was 75% methanol, pH 7.00.

**Figure 6:**
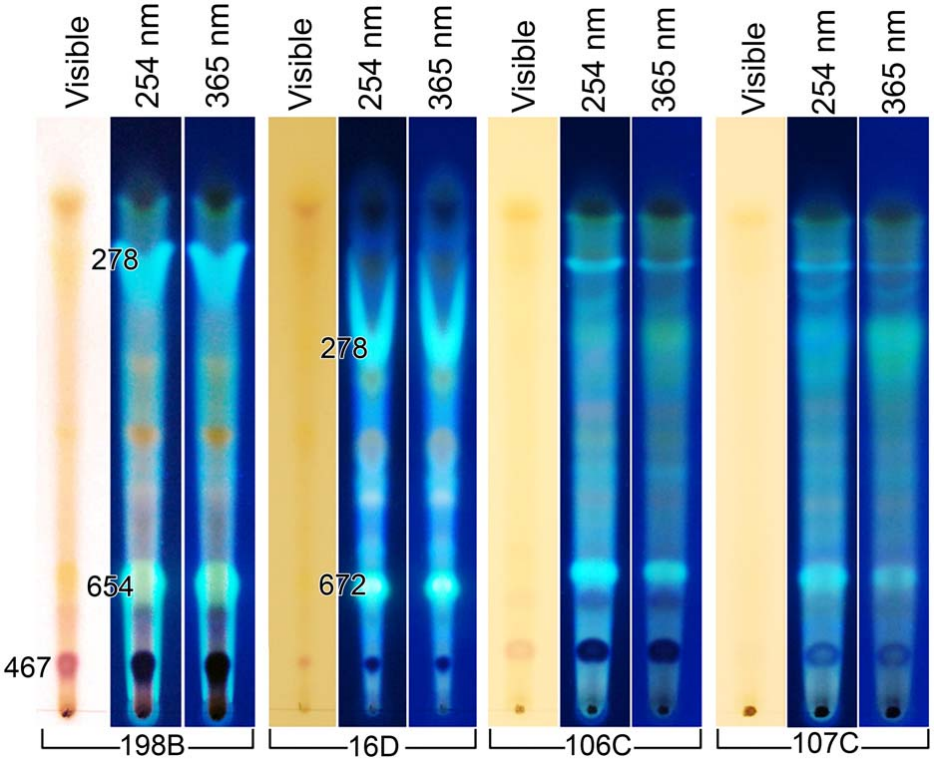
Extracted pigments and fluorescent compounds of *Cryptococcus* using small volumes of methanol. The methanol extracted pigments and fluorescent compounds of *C. gattii*, serotype B, (198B), *C. neoformans* var. *neoformans* (16D), and *C. gattii,* serotype C (106 and 107). The cells were extracted once with a small quantity of methanol. This type of extraction greatly favored obtaining the fluorescent compounds and minimized the extraction of the brown pigments. The figure also suggests that metabolic differences between the *C. gattii* strains exist. The solvent system was 75% methanol, pH 7. The molecular mass for several compounds was determined.

TLC analysis of the *C. gattii* pigments and fluorescent compounds recovered with multiple methanol extractions revealed the production of pink and yellow pigments that were not observed in culture because they were masked by the brown pigments (Fig. 5, first section visible light). Fluorescence at 254 nm and 365 nm (Fig. 5, middle section) and a merged pattern (Fig. 5, last section) revealed that *Cryptococcus* spp. produced multiple pigments and fluorescent compounds after growth on L-tryptophan. In contrast, the TLC results (Fig. 6), of a single 5 ml methanol extraction, removed very little pigment from the yeast cells but extracted a significant amount of the fluorescent compounds. The molecular mass of the fluorescent that were extracted ranged from 278 to 672 (Fig. 6). Additionally, an intracellular water insoluble pink pigment produced by *C. gattii* had a molecular mass of 467 Da (Fig. 6). Alternately, the cryptococcal cells could be extracted with methanol at days 2 or 3, an approach that also maximized the quantity of the fluorescent compounds and minimizes the amount of the pigments (results not shown). The bulk of the pigments were formed in the stationary phase of growth.

**Figure 7:**
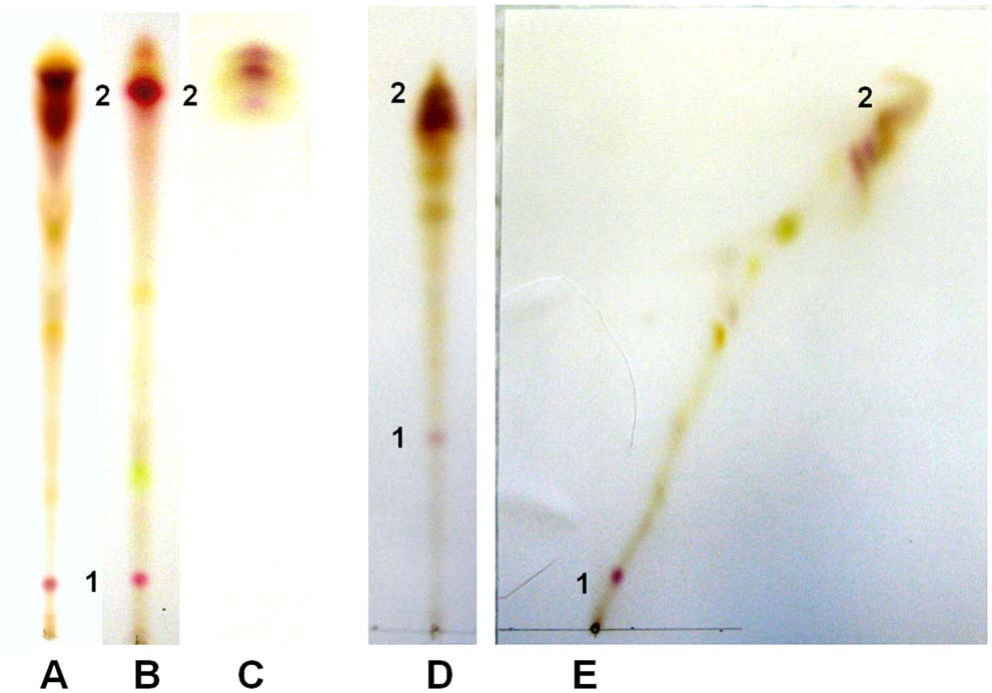
TLC of pink pigments. Columns A and B were extracted pigments from *C. neoformans* var. *neoformans*; pink pigment 1 was water insoluble and pink pigment 2 was water soluble. Column C shows the extracellular water soluble pink pigment 2 produced by *C. neoformans* var. *grubii.* Columns D and E (two dimensional TLC) were the extracted pigments from *C. gattii.* The solvent was 75% methanol, pH 4 to 5. The acid pH solvent developed the pink intracellular water soluble pink pigment, 2. The molecular weight of both the extracellular or intracellular pink pigment, 2, was 535.2 for each *Cryptococcus* spp.

**Figure 8:**
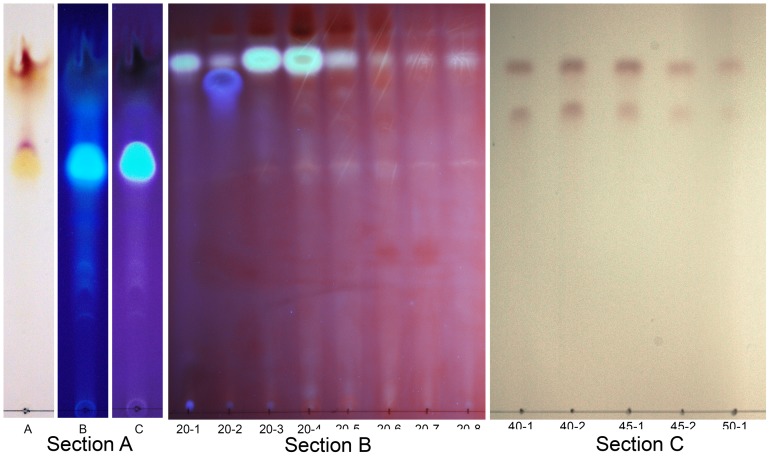
TLC of the pink extracellular pigment. The TLC results of the *C. neoformans* concentrated supernatant under visible light, 254 nm, and 365 nm (Section A). The pink pigment separates into a major band (top) and a minor band (lower) which is located at the top of the tryptophan band (Section A). C 18 Sep-Pak column supernatant separation of fluorescent compounds which were then detected on TLC. The fluorescent compounds including tryptophan (blue fluorescence) were eluted with 20% methanol (section B). Multiple 5 ml 20% methanol fraction were collected. After the 8^th^ tube washing with 20% methanol fluorescent compounds were no longer detected in the fractions. The pink pigment was then eluted off the C18 Sep-Pak column with 40% to 50% methanol (section C). The eluted pink pigment formed two bands.

TLC of the pink water soluble pigments from the three *Cryptococcus* spp. revealed that it consisted of heterogeneous compounds (Fig. 7). Most strains of *C. neoformans* var. *neoformans* and all strains of *C. gattii* required methanol to extract the pink water soluble, molecular mass of 535.2 Da, pigments from the yeast cells (Fig. 7, sections A, B, and D). Two dimensional chromatography was necessary to separate the pink water soluble pigment, molecular mass of 535.2 Da produced by *C. gattii* and some strains of *C. neoformans* var. *neoformans* (Fig. 7, section E). A second water insoluble pink pigment was detected in extracts of *C. gattii* and *C. neoformans* var. *neoformans* (Fig. 7). The TLC separation of the pink pigment, molecular mass of 535.2 Da, from concentrated supernatants is depicted in (Fig. 8A) and after application to a C18 Sep-Pak column (Fig. 8B and C).

### Partial Purification of the Extracellular Pink Pigment

The pink pigments of *C. neoformans* derived from L-tryptophan were compared to the pink pigments produced by *C. albicans* from L, and D-tryptophan by means of TLC. The R_f_ obtained for the pink pigment produced by *C. albicans* from L or D-tryptophan was identical to R_f_ pink pigment produced by *C. neoformans* from L- tryptophan. The pink pigment was estimated to be 90% pure by TLC. The pink pigment migrated as two bands with the top band usually, R_f_ 0.86–0.90, having the highest purity (Fig. 7A, B, and C, Fig. 8C) whereas the bottom band comigrated with another metabolite that was present in the supernatant. The pink pigment separated into these two components on TLC regardless of the solvent system. The pink pigment eluted from a C18 Sep-Pak column also separated into two bands when analyzed by TLC regardless of the solvent system. The molecular mass of the pink pigment was always 535.2 Da for both the major and minor bands of the pink pigment as eluted from TLC plates (Fig. 7 and 8C). The pink pigments that were analyzed by TLC were not fluorescent under a UV Lamp at either 365 or 254 nm. Ninhydrin spray did not react with the extracellular pink pigment indicating that an amino group is not part of the structure of the compound.

### Transmission Electron Microscopy

Melanin accumulates in the cell wall of *Cryptococcus neoformans* [33], [34]. The cell walls and the interior of *C. gattii* were normal in appearance after growth on m-FLTG medium. The cells failed to accumulate electron dense particles in the cell wall after producing brown intracellular pigments from L-tryptophan (results not shown).

### Acid Resistance of L-tryptophan Pigments

*C. gattii* cells that were cultured on m-FLTG medium produced dark brown pigments that were not acid resistant. Hence, tryptophan particles were not formed. In contrast, *C. gattii* and *C. neoformans* var. *grubii* and *C. neoformans* var. *neoformans* produced melanin from L- and D-DOPA media [20]. Melanin particles “ghosts” were recovered after acid treatment [23], [40].

### Differences between Pigmentation from L-tryptophan and DL-, D-, and L-DOPA

Pigmented cells grown on L-tryptophan manifested numerous differences from cells grown on DL-, D-, and L-DOPA. The results obtained using L-tryptophan was identical to the results previously reported comparing D-tryptophan derived pigment to DOPA derived pigments [3]. The most striking difference was that L-tryptophan derived pigments were extracted with 100% alcohol whereas organic solvents were not able to extract melanin [18], [31].

### Requirement for Laccase in Tryptophan-derived Pigment Production

**Figure 9:**
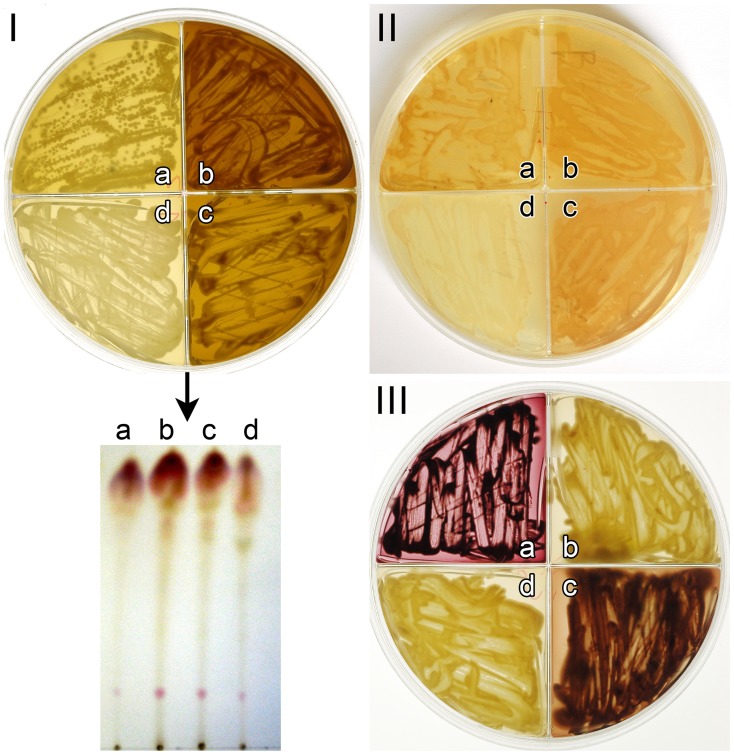
Laccase mutants of Cryptococcus. *C. neoformans* var. *neoformans* grown on m-LTG agar for two weeks (section I), quadrant a (2ETU-C), quadrant b (B3501), quadrant c (JEC21) and quadrant d (2ETU, laccase mutant). The laccase mutant failed to produce the brown pigment. Similar results were obtained when the same strains were cultured on D-tryptophan (m-FDTG agar) (section II). *C. neoformans* var. *grubii* grown on m-FLTG agar for two weeks (section III), quadrant a (H99, *lac_1_* and *lac_2_* positive), quadrant b (MDJ12 (*lac_1_* mutant), quadrant c RPC26 (*lac_2_* mutant), and quadrant d QGC8 (*lac_1_* and *lac_2_* double mutant). When *lac_1_* was deleted, production of the pink pigment was significantly reduced. The TLC illustrated that when the pink pigment was produced intracellularly the laccase mutant of *C. neoformans* var. *neoformans* (2ETU) (column d) produced reduced amounts of the pink pigment.

*C. neoformans* strains deficient in *lac* 1 made no pigments from either L- and D-tryptophan (Fig. 9). *C. neoformans* var. *neoformans lac* 1 mutant failed to produce pigment from L-tryptophan (Fig. 9, section1) and D- tryptophan (Fig. 9, section II). *C. neoformans* var. *grubii lac* 1 mutants failed to produce the pink pigment on m-FLTG agar (Fig. 9 section III). Similar results were obtained on m-LTG agar (results not shown).

## Discussion

Both *C. albicans* and *C. neoformans* were reported to produce a pink extracellular pigment from L-, or DL-tryptophan but we have no information as to the species or serotypes of these cryptococcal isolates [35], [36]. Pink pigment production usually required special lighting. After selecting glycine as an auxiliary nitrogen source (m-LTG medium), normal laboratory lighting resulted in pink pigment production by *Cryptococcus* spp. Our results showed that 100% of the *C. neoformans* var. *grubii* strains, 36% of the *C. neoformans* var. *neoformans* strains, and 86% of the serotype AD strains produced large amounts of an extracellular pink pigment when grown in m-LTG medium. In contrast, *C. gattii* and 64% of *C. neoformans* var. *neoformans* strains produced smaller amounts of the pink water soluble pigment which was retained intracellularly after growth on m-FLTG medium. The pink extracellular pigment produced by *C. albicans* from D- and L-tryptophan [36], [37] and the pink pigment produced from L-tryptophan by *C. neoformans* var. *grubii*, *C. neoformans* var. *neoformans,* and serotype AD as well as the pink water soluble pigments produced intracellularly by *C. gattii* and *C. neoformans* var. *neoformans* were identical. Each had the same molecular mass (535.2 Da) as determined by mass spectrometry. Furthermore, the pink water soluble pigment produced by each of these yeasts had the same pH behavior with respect to the appearance of the pink color. The pink pigment was not produced by either *C. gattii* or *C. neoformans* from D-tryptophan [37].

*Lac1* mutants of *C. neoformans* var. *grubii* and *C. neoformans* var. *neoformans* were unable to produce the extracellular pink water soluble pigment from L-tryptophan except for trace amounts. These results implicated laccase in tryptophan-derived pigment production and suggested that laccase has a broader activity than previously described such that tryptophan is metabolized to a series of both extracellular and intracellular pigments. Additionally, the location of the enzyme laccase was associated with the cell wall of *Cryptococcus*, especially at a neutral pH and glucose starvation [33], [34]. It has been suggested that this enzyme may have a periplasmic or cytosolic location under other conditions such as an acidic pH and/or high carbohydrate concentrations [33]. In our L-and D-tryptophan studies the pH was acidic and the carbohydrate concentration was above starvation levels. These results suggest that when tryptophan was metabolized by *Cryptococcus* laccase was localized in the cytoplasm rather than the cell wall. In this regard, laccase is found in the cytoplasm, vesicles and cell wall [41].

The conservation values for the amino acid sequences of laccase for the *Cryptococcus* spp. ranged from 96% to 80% [42]. The fact that the three *Cryptococcus* spp. (including all 5 serotypes) produced the same pink pigment is consistent with the laccase amino acid homology studies.

*Cryptococcus* spp. and *C. albicans* both produced an identical pink pigment (535.2 Da) but with only the former requiring laccase. Melanin was detected in *C. albicans* in cytoplasmic extracts [43]. Additionally, melanin production by *C. albicans* occurred after 3 to 4 days of incubation in L-DOPA medium [44]. Melanin formation was externalized from the *C. albicans* cells in the form of melanosomes that were loosely bound to the cell wall exterior [44]. Despite these two reports of melanin production by *C. albicans* no laccase-encoding sequences have been detected in the *Candida* genome [44].

*C. gattii* showed the least variation of the *Cryptococcus* spp. since it consistently produced predominantly brown pigments from both D-tryptophan [37], and L-tryptophan (current study).The brown intracellular pigments produced from L-or D-tryptophan by *C. gattii* was distinct from melanin [37]. Cryptococcal melanin is found in the yeast cell wall [34], [38] whereas tryptophan pigments were found to be primarily located in the interior of the cell [37]. Additionally, acid treatment of DOPA-melanized cells results in the formation of melanin particles (“ghosts”) [23], [38] whereas both L- and D-tryptophan pigments were digested by the acid and no ‘ghost’-like particles were formed [37]. A striking difference between the melanin pigments and the L- and D-tryptophan derived pigments was that the former were insoluble in organic solvents while the latter were readily extracted with alcohols. Laccase-derived melanin requires the presence of hydroxyl or amino groups on the aromatic ring features not found in tryptophan [17]–[32]. An important characteristic of melanin is the presence of stable free radical population that gives a distinctive signal by electron paramagnetic resonance (EPR) [45]. The EPR spectrum of the DOPA-derived pigment (melanin) was distinctive and clearly different from the EPR spectrum derived from the tryptophan pigments [37]. Laccase has been associated with the virulence of *Cryptococcus* and the deletion of the enzymes resulted in the loss of its virulence [38], [39].

Preliminary information in the current study indicated that laccase was also required for brown pigment production. *Lac1* mutants of *C. neoformans* var. *neoformans* failed to produce the brown pigments from either L- or D-tryptophan. *Lac1* mutants of *C. gattii* were not available.

The diploid serotype AD was more likely to resemble serotype A than serotype D, at least with respect to pigment production from L-tryptophan (m-LTG) since 86% secreted the pink pigment into the supernatant whereas the results were 100% for *C. neoformans* var. *grubii* (serotype A) and only 36% for *C. neoformans* var. *neoformans* (serotype D).

The metabolism of L-tryptophan and D-tryptophan by *Candida* and *Cryptococcus* species [31], [35], [37] was very different from the tryptophan metabolism reported for *Saccharomyces* species and for bacteria such as *Escherichia coli.* These microorganisms metabolize tryptophan to products such as anthranilic acid, indole, skatole, kynurenine and niacin. *Cryptococcus* produced melanin-like pigments from indole derivatives that contained hydroxyl groups on the heterocyclic rings [28]. *Cryptococcus* species produced tryptophol and indolelactic acid from tryptophan [46] and 3-hydroxyanthranilic acid [26] was found in the supernatant of cryptococcal cultures. In contrast, this study reports the conversion of L-tryptophan by *Cryptococcus* spp. to multiple intracellular and extracellular pigments and fluorescent compounds. The molecular weights of the tryptophan metabolites analyzed in this study ranged from 228 to 672 Da. These results suggest that in *Cryptococcus*, laccase metabolized tryptophan via a synthetase pathway to a series of pigments and fluorescent compounds. Tryptophan was metabolized to a series of indole alkaloid compounds by *Malassezia furfur* and *Candida glabrata* [47], [48]. The pink-brown pigmented compounds produced included pityriarubin A, B, and C by *Malassezia furfur* and pityriarubin C by *C. glabrata.* The molecular mass of the three pitriarubin compounds ranged from 524.5 to 526.6. Fluorescent compounds including pityriacitrin and pityrialactone were also produced from tryptophan by *Malassezia furfur* [49], [50]. These results suggest that the currently described pathways of tryptophan metabolism are incomplete and that there are additional pathways and metabolites that may play important roles in yeast physiology.

Given the ongoing spread of *C. gattii* in North America [3], [9]–[12] and increasing evidence that there are differences in the clinical outcome resulting from cryptococcosis caused by the different Cryptococcal spp. there is an increasing need for simple assays to distinguish between strains. Currently there are only a few biochemical tests that distinguish between *C. gattii, C. neoformans* var. *grubii* and *C. neoformans* var. *neoformans.* Canavanine- glycine-bromthymol blue agar (CGB) or D-tryptophan D-proline agar (m-DTDP) results in the growth of *C. gattii* and the inhibition of *C. neoformans* var. *grubii*, *C. neoformans* var. *neoformans* and serotype AD [37], [51]. *C. gattii* can also be distinguished from *C. neoformans* var. *grubii* and *C. neoformans* var. *neoformans* by either D-proline [52]–[55] or D-tryptophan assimilation tests [52], [53], [56]. *C. gattii* can assimilate these D-amino acids whereas strains of the *C. neoformans* var. *grubii, C. neoformans* var. *neoformans,* and serotype AD lack this ability.

In comparison the separation of *C. neoformans* var. *neoformans* from *C. neoformans* var. *grubii* utilizing biochemical testing has been difficult to achieve. Irokanulo et al. [57] separated the two varieties with creatinine dextrose bromthymol blue thymine agar.

*C. gattii* (100%) and *C. neoformans* var. *neoformans* (64%) produced brown intracellular pigments from L-tryptophan (m-LTG and m-FLTG agars). In this study 64% of the isolates of *C. neoformans* var. *neoformans* were differentiated from *C. neoformans* var. *grubii* by utilizing m-LTG agar in combination with m-DTDP agar [37]. Cryptococcal isolates that produce brown pigments after growth on L-tryptophan medium but failed to grow on m-DTDP agar can be confirmed as *C*. *neoformans* var. *neoformans*. Thus the complete separation of the two *Cryptococcus* varieties cannot be achieved using a combination of L-and D-tryptophan media.

In summary, *Cryptococcus* spp. produced two major types of pigments from L-tryptophan of which one is a water soluble compound of mass 535.2 Da with pink coloration that varies with pH and the other is an organic soluble cell-associated brown pigment. Pigment production was dependent on laccase but neither pigment was a melanin. Although the structure of the pigments remains elusive they appear to be tryptophan-derived molecules. This study optimized the conditions needed for the production of the pink pigment including minimizing the requirement for light. Additionally, the current study found that the pink pigment produced from L-tryptophan was retained intracellularly by *C. gattii* and excreted by *C. neoformans* var. *grubii. C. neoformans* var. *neoformans* varied with some strains producing the pigment intracellularly and others favored extracellular production. In contrast all *Cryptococcus* spp. and serotypes were unable to produce the pink pigment from D-tryptophan [37].This study proved that pigment product from L, and D-tryptophan was distinct. In contrast the metabolism of DOPA (L and D) by *Cryptococcus* was identical regardless of the enantiomorph studied [20]. Finally, the current study showed that the pink pigment produced by both *Cryptococcus* and *Candida* are almost certainly identical since they have the same molecular mass and behave in an identical fashion in reference to pH with respect to the appearance and disappearance of the pink color. This study showed that the production of the pink pigment by *Cryptococcus* and *Candida* involved two different mechanisms since only the former required laccase. Our study clarifies past reports of pigment production by *Cryptococcus* spp. and suggests a need for future studies to establish the physiological role for these compounds in cryptococcal biology and their effects, if any, in pathogenesis.
